# Systematic Review of Randomized Clinical Trials of Acupressure Therapy for Primary Dysmenorrhea

**DOI:** 10.1155/2013/169692

**Published:** 2013-08-18

**Authors:** Hui-ru Jiang, Shuang Ni, Jin-long Li, Miao-miao Liu, Ji Li, Xue-jun Cui, Bi-meng Zhang

**Affiliations:** ^1^Longhua Hospital, Shanghai University of Traditional Chinese Medicine, No. 725 South Wanping Road, Shanghai 200032, China; ^2^Shanghai First People's Hospital, Shanghai Jiaotong University, Shanghai 200080, China

## Abstract

The evidence of acupressure is limited in the management of dysmenorrhea. To evaluate the efficacy of acupressure in the treatment of primary dysmenorrhea based on randomized controlled trials (RCTs), we searched MEDLINE, the Chinese Biomedical Database (CBM), and the Cochrane Central Register of Controlled Trials (CENTRAL) databases from inception until March 2012. Two reviewers independently selected articles and extracted data. Statistical analysis was performed with RevMan 5.1 software. Eight RCTs were identified from the retrieved 224 relevant records. Acupressure improved pain measured with VAS (−1.41 cm 95% CI [−1.61, −1.21]), SF-MPQ at the 3-month followup (WMD −2.33, 95% CI [−4.11, −0.54]) and 6-month followup (WMD −4.67, 95% CI [−7.30, −2.04]), and MDQ at the 3-month followup (WMD −2.31, 95% CI [−3.74, −0.87]) and 6-month followup (WMD −4.67, 95% CI [−7.30, −2.04]). All trials did not report adverse events. These results were limited by the methodological flaws of trials.

## 1. Introduction

Primary dysmenorrhea is defined as the occurrence of painful menstrual cramps of uterine origin in women during menstruation without any evident pathology [1]. It is characterized by crampy pelvic pain with pain radiating to the lower back or anterior thigh, nausea, vomiting, diarrhoea, headache, fatigue, nervousness, and dizziness beginning shortly before or at the onset of menses and lasting one to three days [2]. The prevalence of dysmenorrhea is highest in adolescent women, with estimates ranging from 40 to 50 percent [3]. It is the leading cause of recurrent short-term school absenteeism in adolescent girls in the United States [3, 4]. Most adolescents self-medicate with over-the-counter medicines, and few consult a physician about dysmenorrheal [5, 6].

Therapies for primary dysmenorrhea include pharmacological, nonpharmacological, and surgical approaches; however, many of the standard treatments have not been well studied. The recommendations reflect a balance between the available evidence and an assessment of benefit, harm, and cost [7]. Nonsteroidal anti-inflammatory drugs (NSAIDs) are the best-established initial therapy for dysmenorrheal [8]. They have a direct analgesic effect through inhibition of prostaglandin synthesis, and they decrease the volume of menstrual flow. Surgical approaches may be used, but the evidence for nerve interruption in the management of dysmenorrhea is limited [9]. Complementary and alternative medicines, including supplements, herbal remedies, physical treatments, are choices of these patients [8, 10–13]. As a noninvasive technique, acupressure relieves pain by pressing the special acupoints with fingers or thumbs [14]. However, there is no convincing evidence in the treatment of primary dysmenorrhea. It is necessary to evaluate the efficacy of body acupressure in the treatment of primary dysmenorrhea when compared with a placebo, no treatment, or conventional medical treatment based on randomized controlled trials (RCTs). 

## 2. Methods

### 2.1. Search Strategy

The literature search was performed on MEDLINE, the Chinese Biomedical Database (CBM), and the Cochrane Central Register of Controlled Trials (CENTRAL) databases from inception until March 2012 to identify pertinent RCTs. We combined acupressure-related terms (Chih Ya OR Zhi Ya OR Shiatzu OR Shiatsu OR Acupressure) with dysmenorrhea-related terms (Painful Menstruations OR Painful Menstruation OR Menstruations, Painful OR Menstruation, Painful OR Pains, Menstrual OR Menstrual Pains OR Menstrual Pain OR Pain, Menstrual OR Dysmenorrhea OR Dysmenorrheas). In addition, a manual search was performed of the reference lists of studies and reviews. 

### 2.2. Study Selection

All RCTs meeting the following criteria were included in the review. The patients should be primary dysmenorrhea (self-reported pain) during the majority of the menstrual cycles or for three consecutive menstrual cycles with moderate to severe primary dysmenorrhea. The trial was excluded if the patients were diagnosed with secondary dysmenorrhea. The RCT should be involved acupressure for the treatment of primary dysmenorrhea. The acupressure intervention was compared to placebo control, rest, pharmacological management, or other conventional treatments. The primary outcome was pain relief measured by a visual analogue scale (VAS) or other validated scales. Secondary outcomes included overall improvement measured by Short-Form McGill Pain Questionnaire (SF-MPQ), Menstrual Distress Questionnaire (MDQ), quality of life measured by a validated scale, for example, the Short Form (SF) 36, and adverse effects measured as incidence of side effects or other types of side effects.

### 2.3. Data Collection and Analysis

Two reviewers independently selected articles. The titles and abstracts of articles found in the search were screened by Hui-ru Jiang and Shuang Ni, who discarded trials that were clearly not eligible. Trial was selected by two review authors (Hui-ru Jiang and Shuang Ni). Hui-ru Jiang and Jin-long Li independently extracted data using the designed form. All discrepancies were resolved by discussion. Two review authors (Ji Li and Xue-jun Cui) checked and entered data into Review Manager (RevMan 5.1).

Risk of bias was assessed independently by two review authors (Hui-ru Jiang and Shuang Ni) with the criteria in the Cochrane Handbook for Systematic Reviews of Interventions 5.1.0 [23]. Sequence generation, allocation concealment, blinding (or masking), incomplete data assessment, selective outcome reporting, and other sources of bias were assessed with three potential responses: yes, no, and unclear (Table 1). Disagreements between review authors were resolved by discussion or with a third author (Bi-meng Zhang).

Statistical analysis was performed with RevMan 5.1 software. For dichotomous data, results for each study were expressed as Peto odds ratios (OR) with corresponding 95% confidence intervals (CI) using the Mantel-Haenszel method. We expressed continuous data as weighted mean differences (WMD) with 95% CI or as standardized weighted mean differences (SMD) if outcomes were conceptually the same but measured in different ways in the different trials.

If there were “multi-arm” studies, for example, there are two acupressure groups with a common control group or two control intervention groups such as a placebo group and a standard treatment group, and data from both treatment arms were combined into one group. For studies with a placebo control and no treatment control group, the shared intervention was divided evenly between groups as described in the Cochrane Handbook [23]. 

We included a more formal chi^2^ test. A low *P* value (or a large chi^2^ statistic relative to its degree of freedom) provided evidence of heterogeneity of intervention effects (variation in effect estimates beyond chance). We measured inconsistency across trials in the meta-analysis using the *I* ^2^ statistic described in the Cochrane Handbook for Systematic Reviews of Interventions [23]. We planned to investigate potential biases of publication using the funnel plot or other analytical methods [24]. In the presence of significant heterogeneity, we aimed to examine the causes by subgroup analysis and also sensitivity analysis. If subgroup analysis failed to explain the heterogeneity, data were analyzed with the random-effects model. 

## 3. Results

### 3.1. Description of Included Studies

The search retrieved 224 potentially relevant records, including 86 trials from PubMed, 125 from CBM, 2 from CENTRAL, and 11 trials from manual search. 177 records were excluded by screening the titles and abstracts, including 8 duplicates. The remaining 47 full-text articles were retrieved for additional scrutiny, of which 39 proved ineligible because of no mention of randomization or quasirandomised trials. Thus 8 trials were included in the current review. The types of control interventions, and data extraction for these 8 studies are depicted in Table 2. 

All the 8 trials used a parallel design, including 800 patients [15–22]. Six trials had two study groups [15, 16, 18, 19, 21, 22]. One trials had three groups [20] and one trials had four arms [17]. Comparative and control groups varied. Six trials used no treatment controls, including placebo, rest, and waiting control [15–19, 22]. Two are single-blind clinical trials, in which participants were treated with placebo acupressure [18, 19]. Comparisons with medication using Ibuprofen were used in two trials [20, 21].

The research location of 3 trials was in China (two in Taiwan, one in Hong Kong, and none in Chinese Mainland), one in the USA, and four in Iran. Among these trials, the largest sample size was 216 [20], the smallest sample size was 30 [19], and the average sample size was 100. The data in each trial were analyzed individually with special software (RevMan 5.1).

Fixed sets of acupressure points were used in all trials. Patients received acupressure for one menstrual cycle in two studies [19, 20], two menstrual cycles in three studies [16, 18, 21], and three menstrual cycles in three studies [15, 17, 22]. VAS scales were used in six trials to assess pain relief [16–20, 22]. An assessment of pain and other menstrual symptoms was undertaken in the remaining two studies with Andersch and Milsom scale [15], and Descriptive Numeric Rating Scale of Pain Intensity and Dysmenorrhea Symptom Intensity and Distress Inventory [21].

### 3.2. Risk of Bias in Included Studies

**Figure 1:**
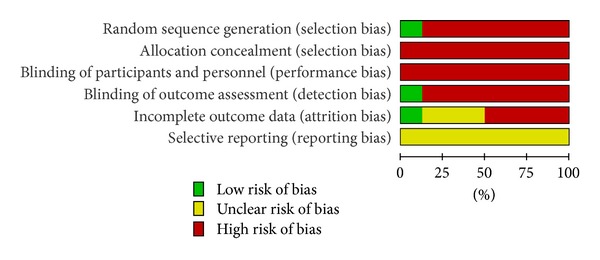
Risk of bias graph: review authors' judgements about each risk of bias item presented as percentages across all included studies.

**Figure 2:**
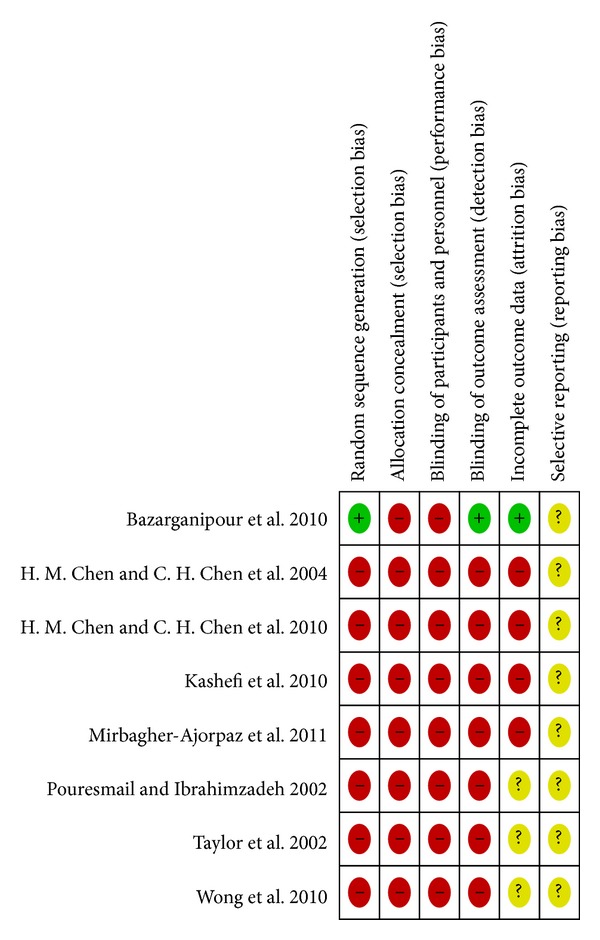
Risk of bias summary: review authors' judgements about each risk of bias item for each included study.

See Figures 1 and 2 for a graphical summary of the risk of bias assessments of the included studies made by authors based on the risk of bias domains. No trial was at a low risk of bias on all domains.

Using the Cochrane criteria, we rate the risk of bias. Only one of 8 studies had a low risk of selection bias, detection bias, and attrition bias [15], and the sequence was generated by means of a table of random numbers. We found high risks of bias in the included studies to be failure to describe or use appropriate adequate generation of randomisation sequence (7/8), concealment of allocation (8/8), and lack of effective blinding procedures (observer (7/8); patient (8/8); care provider (8/8)). We acknowledge that it is difficult to blind the patient and impossible to blind the care provider in acupressure treatments. 

### 3.3. Effects of Interventions

**Figure 3:**
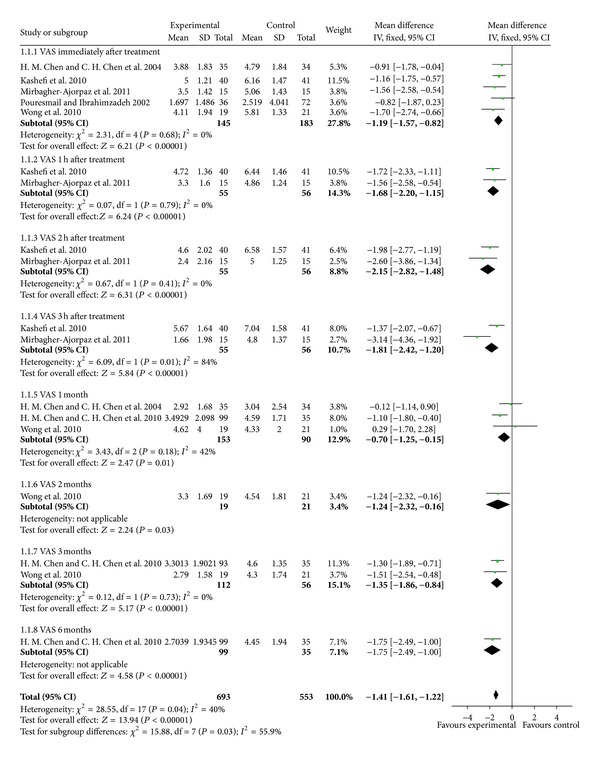
Acupressure versus placebo acupressure or rest control for pain relief.

**Figure 4:**
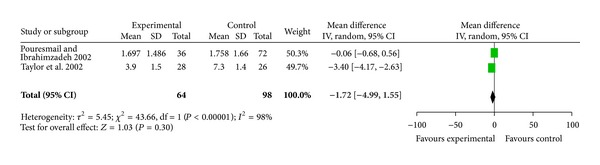
Acupressure versus pharmacologic treatment for pain relief.

Compared with placebo acupressure or rest control, acupressure improved pain measured with VAS (−1.41 cm 95% CI [−1.61, − 1.21]). VAS was less immediately after treatment (WMD −1.19 cm, [−1.57, − 0.82]; 5 trails, 145 participants), 1 h after treatment (WMD −1.68 cm, 95% CI [−2.20, − 1.15]; 2 trails, 55 participants), 2 h after treatment (WMD −2.15 cm, 95% CI [−2.82, − 1.48]; 2 trails, 55 participants), and 3 h after treatment (WMD −1.81 cm, 95% CI [−2.42, − 1.20]; 2 trails, 55 participants), with substantial statistical heterogeneity (*I* ^2^ = 84%, Figure 3). At the end of one, two, three, and six months, the WMD in pain outcomes were −0.70 cm (95% CI [−1.25, − 0.15]; 3 trails, 195 participants), −1.24 cm (95% CI [−2.32, − 0.16]; 1 trail, 19 participants), −1.35 cm (95% CI [−1.86, − 0.84]; 2 trails, 112 participants), and −1.75 cm (95% CI [−2.49, − 1.00]; 1 trail, 99 participants). Two trials compared acupressure with Ibuprofen and found no improvement in pain relief (WMD −1.72 cm, 95% CI [−4.99, 1.55]; 64 participants, Figure 4). 

**Figure 5:**
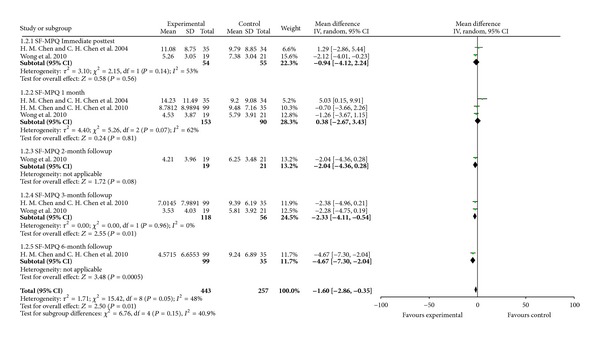
Acupressure versus placebo acupressure or rest control for menstrual symptoms (SF-MPQ).

**Figure 6:**
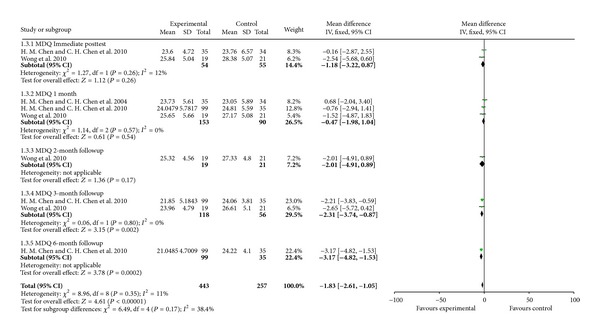
Acupressure versus placebo acupressure or rest control for menstrual symptoms (MDQ).

For improvement in symptoms, acupressure did not improve SF-MPQ compared with placebo acupressure or rest control at immediate posttest in two studies with 99 participants (WMD −0.94, 95% CI [−4.12, 2.24]) and 1-month followup in three studies with 243 participants (WMD 0.38, 95% CI [−2.67, 3.43]) and 2-month followup in one study with 40 participants (WMD −2.04, 95% CI [−4.36, 0.28]) but improved at the 3-month followup in two studies with 174 participants (WMD −2.33, 95% CI [−4.11, − 0.54]) and 6-month followup in one study with 134 participants (WMD −4.67, 95% CI [−7.30, − 2.04]) (Figure 5). Acupressure did not improve menstrual symptoms (MDQ) compared with placebo acupressure or rest control at immediate posttest in two studies with 99 participants (WMD −1.18, 95% CI [−3.22, 0.87]), 1 month followup in three studies with 243 participants (WMD −0.47, 95% CI [−1.98, 1.04]) and 2-month followup in one study with 40 participants (WMD −2.01, 95% CI −2.01 [−4.91, 0.89]) but improved at the 3-month followup in two studies with 174 participants (WMD −2.31, 95% CI [−3.74, − 0.87]) and 6-month followup in one study with 134 participants (WMD −3.17, 95% CI −3.17 [−4.82, − 1.53]) (Figure 6).

Two trials reported this outcome as a dichotomous variable and were not included in meta-analysis. Bazarganipour et al. reported that the severity of dysmenorrhea was significantly different in the fourth cycle (*U* = 2377, *P* < 0.001) [15]. Taylor et al. reported the reduction of menstrual pain as being significantly better in the case of worst menstrual pain (WMD −3.40, 95% CI [−4.17, − 2.63]), menstrual pain symptom intensity (WMD −4.20, 95% CI [−5.33, − 3.07]), and in pain medication consumption (WMD −6.40, 95% CI [−10.49, − 2.31]) in an acupressure plus pharmacologic treatment group than in a pharmacologic treatment group alone at the end of the second circle [21]. 

## 4. Discussion

There are only eight trials assessing the role of acupressure in the management of primary dysmenorrhoea. Eight studies and data from 800 participants were included in the systematic review. Six studies examined the efficacy of acupressure using placebo controlled designs. There was some limited evidence of acupressure showing a benefit in relation to the primary outcomes of pain relief (VAS) and reduced menstrual symptoms. There was an improvement in pain relief compared with placebo or rest control (WMD −1.41 cm, 95% CI [−1.61, − 1.21]) and compared with Ibuprofen (WMD −2.07 cm, 95% CI [−4.27, 0.12]). Acupressure reduced menstrual symptoms compared with placebo or rest control (SF-MPQ, WMD −1.60, 95% CI [−2.86, − 0.35]; MDQ, WMD −1.83, 95% CI [−2.61, − 1.05]). The majority of trials did not report on adverse events. These results were limited by the methodological flaws of trials.

There are three other reviews of acupuncture-related therapies for primary dysmenorrhoea [25–27]. Cho and Hwang included 4 trials, suggesting that acupressure alleviates menstrual pain [25]. Yang et al. identified 3 trials of body acupressure and 4 trials of auricular acupressure, which were excluded in our review [26]. Smith CA found that there was an improvement in pain relief from acupressure compared with a placebo control (WMD −0.99, 95% CI −1.48 to −0.49), and in one trial acupressure reduced menstrual symptoms compared with a placebo control (WMD −0.58, 95% CI −1.06 to −0.10) [27]. Cho and Hwang and Yang et al. both included other modalities of TCM and included trials for which we were unable to ascertain the randomisation details [25, 26]. Both reviews found promising evidence for the use of acupuncture to treat primary dysmenorrhoea compared with pharmacological medicine or Chinese herbal medicine. These findings from the three reviews were influenced by the methodological flaws of the trials.

We noted low quality evidence of all the included eight studies suggesting the limited benefit should be interpreted with caution. The completeness and applicability of the evidence is limited as from the 8 included trials, only one reported meaningful data and seven were at a high or unclear risk of bias on all domains. 

Because of low methodologic quality and small sample size, there is no convincing evidence for acupuncture in the treatment of primary dysmenorrhea. Well-designed RCTs with rigorous methods of randomisation, and adequately concealed allocation are needed.

## Figures and Tables

**Table 1:** The Cochrane Collaboration's tool for assessing risk of bias.

Random sequence generation	
Low risk of bias	The investigators describe a random component in the sequence generation process such as referring to a random number table and using a computer random number generator
High risk of bias	The investigators describe a nonrandom component in the sequence generation process. Usually, the description would involve some systematic, nonrandom approach, for example, sequence generated by odd or even date of birth and sequence generated by some rule based on date (or day) of admission
Unclear risk of bias	Insufficient information about the sequence generation process to permit judgement of “Low risk” or “High risk”

Allocation concealment	

Low risk of bias	Participants and investigators enrolling participants could not foresee assignment because one of the following, or an equivalent method, was used to conceal allocation: central allocation (including telephone, web-based, and pharmacy-controlled randomization); sequentially numbered drug containers of identical appearance
High risk of bias	Participants or investigators enrolling participants could possibly foresee assignments and thus introduce selection bias, such as allocation based on using an open random allocation schedule (e.g., a list of random numbers); assignment envelopes were used without appropriate safeguards (e.g., if envelopes were unsealed or nonopaque or not sequentially numbered)

Blinding of participants and personnel	

Low risk of bias	Anyone of the following: no blinding or incomplete blinding, but the review authors judge that the outcome is not likely to be influenced by lack of blinding; Blinding of participants and key study personnel ensured and unlikely that the blinding could have been broken.
High risk of bias	No blinding or incomplete blinding, and the outcome is likely to be influenced by lack of blinding; blinding of key study participants and personnel attempted but likely that the blinding could have been broken

**Table 2:** Characteristics of clinical trials of acupressure therapy for primary dysmenorrhea.

Study ID	Location	N*	Mean age (SD)*	Intervention	Control	Outcome
Bazarganipour et al. 2010 [15]	Iran	88/84	20.01 (1.01)/19.92 (0.87)	AP, Taichong, 2 minutes, continued for 20 minutes	Placebo point	Andersch and Milsom scale

H. M. Chen and C. H. Chen 2004 [16]	Taiwan, China	35/34	18.06 (1.28)/17.54 (1.54)	AP, once a day for bleeding (4–6 weeks); Sanyinjiao	Waiting list control	MDQ, SF-MPQ, VAS for pain, VAS for anxiety

H. M. Chen and C. H. Chen 2010 [17]	Taiwan, China	30/33/36/35	16.75 (1.36)/16.83 (1.79)/16.88 (2.09)/16.77 (1.19)	AP, Zusanli, Hegu, Sanyinjiao, for 20 minutes	Rest, for 20 minutes	MDQ, SF-MPQ, VAS for pain, VAS for anxiety

Kashefi et al. 2010 [18]	Iran	40/41	NA	AP, Sanyinjiao	Placebo AP	VAS, SF-MPQ

Mirbagher-Ajorpaz et al. 2011 [19]	Iran	15/15	22 (1.6)/22 (2.64)	AP, Sanyinjiao	Placebo AP, Sanyinjiao	VAS

Pouresmail and Ibrahimzadeh 2002 [20]	Iran	72/72/72	NA	AP, once per 2 days from 24 h prior to bleeding (3 months); Hegu, Daheng, Zusanli, Sanyinjiao, Taichong	(a) Ibuprofen 9 tablets for 3 days; (b) Sham AP (nonacupoints)	Andersch and Milsom scale, VAS

Taylor et al. 2002 [21]	USA	31/27	30.7 (6.2)/32.7 (5.5)	AP plus Ibuprofen, device placed from day one of bleeding for 3 days (two menstrual cycles)	Ibuprofen	Worst menstrual pain, menstrual symptom intensity, pain medication consumption

Wong et al. 2010 [22]	Hong Kong, China	19/21	22 (0.082)/21.57 (0.746)	SP6 acupressure	Rest	VAS, SF-MPQ, SF-MDQ

Appendix. *Intervention group/control group; AP: acupressure; VAS: visual analogue scale; SF-MDQ: Short-Form Menstrual Distress Questionnaire; SF-MPQ: Short-Form McGill Pain Questionnaire; NA: not available.
